# Description of the male of *Xystromutillabucki* Suárez, 1960 (Hymenoptera, Mutillidae), including new information on the biology of the genus

**DOI:** 10.3897/zookeys.1011.60944

**Published:** 2021-01-18

**Authors:** Roberto A. Cambra, Caroline Nepomuceno Queiros, Jean P. Alves De Deus, Kevin A. Williams, Pedro R. Bartholomay, Jucélia Iantas, Michele C. Nether, Kenji Nishida, Yostin J. Añino, Daisy Saavedra, Maria L. Tunes Buschini

**Affiliations:** 1 Museo de Invertebrados G. B. Fairchild, Estafeta Universitaria Apartado 00017, Universidad de Panamá, Panamá 0824, Panama Universidad de Panamá Panamá Panama; 2 Laboratório de Biologia e Ecologia de Vespas e Abelhas, Universidade Estadual do Centro-Oeste, Rua Simeão Camargo Varela de Sá 03, Vila Carli, 85040-080, Guarapuava, Paraná, Brazil Universidade Estadual do Centro-Oeste Paraná Brazil; 3 Plant Pest Diagnostics Center, California Department of Food & Agriculture, 3294 Meadowview Road, Sacramento, CA 95832, USA Plant Pest Diagnostics Center, California Department of Food & Agriculture California United States of America; 4 Instituto Nacional da Mata Atlântica, Av. José Ruschi n°4, Santa Teresa, Espírito Santo, Brazil Instituto Nacional da Mata Atlântica Espírito Santo Brazil; 5 Centro Universitário de União da Vitória – UNIUV – Avenida Bento Munhoz da Rocha Neto, 3856,84600-000, União da Vitória (PR), Brazil Centro Universitário de União da Vitória União da Vitória Brazil; 6 Universidade Federal do Paraná, Campus Pontal do Paraná, Centro de Estudos do Mar Avenida Beira-mar, s/n CEP: 83255976 Pontal do Sul, Pontal do Parana, Paraná, Brazil Universidade Federal do Paraná Paraná Brazil; 7 Collaborator, Museo de Zoología, Universidad de Costa Rica; and Estación Biológica Monteverde (EBM), Monteverde, Puntarenas, Costa Rica Universidad de Costa Rica Puntarenas Costa Rica

**Keywords:** *Auplopus*, diapause, Neotropical, Pepsinae, *Pseudomethoca*, seasonal abundance, Sphaeropthalminae, spider wasps, velvet ants

## Abstract

The male of *Xystromutillabucki* Suárez, 1960 is described and associated with the female based on couples reared from trap-nests occupied by *Auplopussubaurarius* Dreisbach, 1963 (Hymenoptera: Pompilidae). Information on the diapause of *X.bucki* and Pseudomethocanr.chontalensis (Cameron, 1895) (Hymenoptera: Mutillidae) is presented. Seasonal and annual variation in the abundance of *X.turrialba* Casal, 1969 are also given.

## Introduction

*Xystromutilla* André, 1905 (Mutillidae: Sphaeropthalminae) belongs to the tribe Sphaeropthalmini (Brothers and Lelej 2017). This Neotropical genus has 14 described species (Bartholomay et al. 2019; Pagliano et al. 2020), two from Central America and 12 from South America, with only *X.turrialba* Casal, 1969 and *X.carpenteri* Cambra & Quintero, 2004 known from both sexes (Cambra and Quintero 2004). Morato (1994) mentioned rearing male and female specimens of *X.asperiventris* André, 1905 from the same trap nests, but this male has not yet been described, although specimens were used by Brothers and Lelej (2017) in compiling the characters of *Xystromutilla*.

Four species of *Xystromutilla* have known hosts; three of these attack solitary aculeate wasps and one attacks a solitary bee. André (1906) mentioned a specimen of *X.aequatorialis* (André, 1906) with a label indicating it was a parasite of *Melitomataurea* (Say) (as *Entechniataurea* Say). Morato (1994) reared *X.asperiventris* André, 1905 from Trypoxylon (Trypoxylon) nitidum F. Smith, 1856, Trypoxylon (Trypargilum) lactitarse de Saussure, 1867, Trypoxylonaff.unguicorne Richards, 1934 (Crabronidae), and *Podiumrufipes* Fabricius, 1804 (Sphecidae); Rodríguez and Matías (1996) recorded *X.turrialba* from *Trypoxylon* sp. and *Podium* sp.; and Cambra and Quintero (2004) mentioned that *X.hansoni* Cambra & Quintero, 2004 was reared from a species of Eumeninae (Vespidae).

In this paper, we present the male description and host association for *X.bucki* Suárez, 1960. Information on diapause for Neotropical Mutillidae is provided, as well as seasonal and annual variation in the abundance of *X.turrialba*.

## Materials and methods

The study of *Xystromutillabucki* Suárez, 1960 was carried out from August 2018 to August 2019 in the municipality of Guarapuava, state of Paraná (PR), southern Brazil. Information on the study site and sampling methods with trap-nests were discussed in Cambra et al. (2017).

The study site for flight seasonality of *Xystromutillaturrialba* Casal, 1969 was the field station of the Smithsonian Tropical Research Institute (**STRI**) on Barro Colorado Island (BCI). Information on the study site and sampling methods with Malaise traps were discussed in Cambra et al. (2018).

Photographs of genitalia were made with an Olympus Stylus digital camera using an Olympus BX53F stereomicroscope, with further image processing done using ArcSoft PhotoStudio. The genitalia were stored in a glass vial and placed on the specimen pin. Measurements of the male specimen were made with a calibrated micrometer scale attached to an ocular lens of the stereomicroscope.

The specimens of *Xystromutillabucki* were identified by authors K.A.W., P.R.B. and R.A.C., while the specimens of *Auplopussubaurarius* Dreisbach, 1963 by R.A.C. and Eduardo Fernando dos Santos. The specimens examined are deposited in Museo de Invertebrados G. B. Fairchild, University of Panama, Panama (**MIUP**) and in the entomological collection of Laboratório de Biologia e Ecologia de Vespas e Abelhas, Universidade Estadual do Centro-Oeste, Guarapuava (PR), Brazil (UNICENTRO). The specimens of Pseudomethocanr.chontalensis are deposited in Museo de Zoología, Universidad de Costa Rica, San José, Costa Rica(**MZUCR**).

## Results

### Taxonomy

#### Xystromutilla bucki

Taxon classificationAnimaliaHymenopteraMutillidae

Suárez, 1960

A4C903A6-E70B-50A5-B5AB-C76BE30B9968

Figs 1–8

Xystromutilla bucki Suárez, 1960: 453–455, ♀, holotype, Porto Alegre, [Rio Grande do Sul], Brasil, 19.iii.1952, P. Buck (Colección Suárez; now in Museo Nacional de Ciencias Naturales, Madrid, Spain).

##### Diagnosis.

**Figures 1–8.**
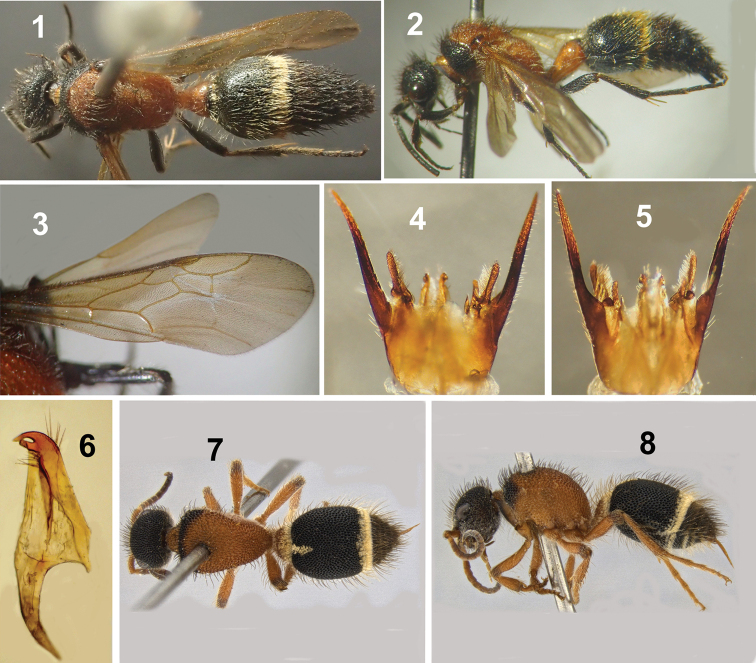
*Xystromutillabucki***1–6** male: **1** dorsal view **2** lateral view **3** right wings **4** genitalia, dorsal view **5** genitalia, ventral view **6** penis valve, lateral view **7, 8** female: **7** dorsal view **8** lateral view.

**Male** (Figs 1–6). This species can be recognized by its unique coloration, wherein the meso-metathorax, propodeum and first metasomal segment are orange-red; wings subhyaline. The following morphological characters are also useful for diagnosis: head with simple setae; mandible ventrally with a strong basal tooth; sternum 1 without a spine; hypopygium posterior margin with a small denticle medially; paramere almost straight and cuspis finger-shaped. Other described males of *Xystromutilla* have black integument, fore wings partly or totally fuscous, sternum 1 with a basal spine, hypopygium with medial spine on the apical margin, paramere lyre-shaped and cuspis elongate spoon-shaped. These morphological characters are not present in males of *X.bucki*. **Female** (Figs 7, 8). Head, pronotum and metasomal segment 1 orange-red, rest of metasoma black; head with simple setae only; humeral angles of pronotum rounded, not carinate; integument of basal half of tergum 2 without carinae.

##### Description.

**Male** (hitherto unknown). (Figs 1, 2). ***Body length*.** 10.1 mm.

***Body Color*.** Integument black, except apical half of mandible, meso-metathorax, propodeum and first metasomal segment orange-red; tibial spurs white; wings subhyaline, without infuscated area; head, pronotum, mesoscutum and scutellum with long, semierect simple black setae; meso-metapleura and propodeum mostly with long, semierect simple pale white setae; fore legs mostly with simple black setae, mid and hind legs mostly with simple pale white setae and few black setae; metasomal segments one and two with long semierect simple pale white setae, posterior margin of metasomal segment 2 with dense and decumbent band of plumose white setae; metasomal segments 3 to 7 with long, semierect black setae, posterior margins of metasomal segments 3 and 4 with decumbent plumose black setae mostly hidden by simple setae.

***Head*.** Rectangular in dorsal view, frons, vertex and gena with medium-sized, very close punctures; distance between eye margin and lateral ocellus 2.77 × as long as diameter of ocellus; flagellomere I 1.9 × pedicel length; flagellomere II 2.2 × pedicel length; clypeus bidentate medially on anterior margin; mandible apically obliquely tridentate, ventrally with a strong tooth near base.

***Mesosoma*.** Pronotum, mesoscutum, scutellum and mesopleuron with medium-sized, contiguous punctures, metapleuron impunctate; tegula glabrous, except anterior and inner margins setigerously punctate; propodeum totally reticulate; notaulus incomplete, not reaching anterior margin of mesoscutum; scutellum convex; fore wing (Fig. 3) with two submarginal cells; coxae without denticle, tubercle or carina.

***Metasoma*.** First segment petiolate, tergum 1 dorsal face 1.47 × as long as wide; tergum 1–2 and sternum 2 and 7 mostly with medium-sized close punctures; metasomal segments 3–6 with small, close punctures; tergum 7 basal half with small punctures, apical half mostly without punctures; sternum 1 without a spine near base, with two longitudinal carinae diverging posteriorly; hypopygium posterior margin not straight, with a small denticle medially.

***Genitalia*.** Parameres (Figs 4, 5) almost straight, not lyre-shaped; basal half in lateral view broad, distal half gradually narrowing toward apex and slightly curved upwards, ending in a sharp point; dorsal surface with five long setae at end of basal broad half; very short sparse setae elsewhere; digitus and cuspis (Figs 4, 5) finger-shaped, digitus with inconspicuous and sparse setae, cuspis laterally flattened with dense long setae on inner surface; penial valve (Fig. 6) with an apical tooth and preapical projection with blunt apex, apical dorsal edge with five long setae and near to base of preapical projection with six long setae.

##### Material examined.

Brazil: **Santa Catarina**: Porto União, nest 371 (1) (5 mm hole diameter), 13.i.2012–14.ii.2012, J. Iantas, 1 ♀ (MIUP) (reared from nest of *Auplopussubaurarius* Dreisbach, 1963). **Rio Grande do Sul**: Cambara do Sul, Itaimbezinho, 10.iii.2000, col. R. da Cunha, 1 ♀ (MIUP); Guaiba, 116 – km 307, col. F.V. Borges: 15.i.1998, 1 ♂ (MIUP); 30.ix.1999, 1 ♂ (MIUP); São Francisco de Paula, CPCN Pro-Mata, col. B. Harter: 4.viii.1997, 1 ♂ (MIUP); 6.i.1998, 1 ♂ (MIUP). **Paraná**: Turvo, 25°01'55"S, 51°31'53"W, col. M.C. Nether: 22.xii.2012, 2 ♂ (nest 223) (MIUP); 22.ii.2013, 1 ♂ (nest 552) (MIUP); Guarapuava, 25°21'55"S, 51°27'58"W, 24.i.2013, col M.C. Nether, 1 ♂ (nest 447) (MIUP); Guarapuava, 25°24'09.7"S, 51°24'45.5"W, 11.iii.2019, col. C. Queiros and J. De Deus, 6 ♂, 1 ♀ (nests 353, 460) (UNICENTRO); Guarapuava, 25°39'S, 51°42'W, 10.v.2019, col. C. Queiros and J. De Deus, 1 ♀ (nest 365) (UNICENTRO)

##### Distribution.

Brazil (Paraná, Rio Grande do Sul, Santa Catarina).

**Biology.** This is the first record of Pompilidae as a host of *Xystromutilla*. In the Araucaria forest fragments, 66 trap-nests of *Auplopussubaurarius* Dreisbach were examined. Of these nests, *X.bucki* parasitized three of them, all of which were in bamboo, 1.3 cm in diameter and 18.2 cm in length. One of the nests had nine cells, six of which were parasitized by *Xystromutilla* (five males and one female emerged) and two by *Photocryptus* sp. (Ichneumonidae: Cryptinae). The other two parasitized nests contained one cell each, from which emerged a male and a female. Therefore, six males and two females emerged in total from the nests. The males were larger bodied with average head width 0.3 mm (n = 6; SD = 0.01 mm) and the females 0.2 mm (n = 2; SD = 0.06 mm).

We found in *Xystromutillabucki* that the average time between nest collection and adult wasp emergence, for seven of the eight specimens reared, was 265 days (n = 7; SD = 4.2 days), with immatures exhibiting diapause at the prepupal stage (6 males, 1 female). Only one female (from the one-celled nest) did not enter into diapause, but rather emerged 11 days after nest collection.

#### Xystromutilla turrialba

Taxon classificationAnimaliaHymenopteraMutillidae

Casal, 1969

9CE9774A-3CC2-5793-8146-E81A2287BA17

Xystromutilla turrialba Casal, 1969, Physis 29: 47, holotype female, Turrialba, Costa Rica, USNM.

##### Material examined.

(81 specimens, MIUP): Panamá, Barro Colorado Island: iv.2001, 15 males; v.2001, 5 males; v.2002, 2 males; vi.2002, 5 males; vii.2002, 1 male; iii.2003, 14 males, 1 female; iv.2003, 27 males, 1 female; v.2003, 5 males; ii.2004, 2 males; iv.2004, 2 males; v.2004, 1 male.

##### Distribution.

Honduras, Nicaragua, Costa Rica, Panama (Casal 1969; Cambra and Quintero 2004).

##### Seasonal and annual abundance.

**Figure 9.**
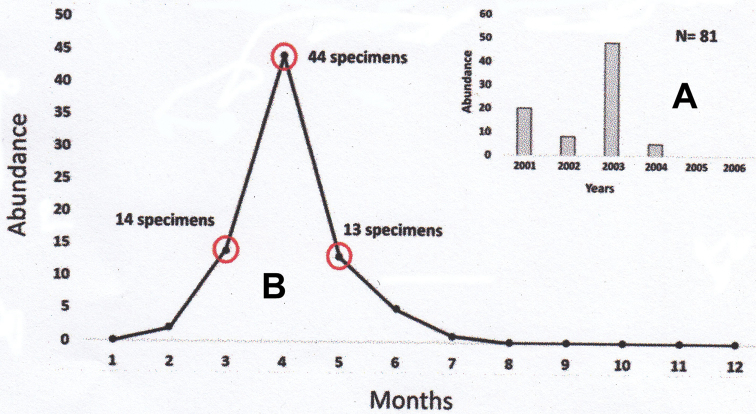
**A** total *Xystromutillaturrialba* specimens captured by year (2001–2006) **B** total *Xystromutillaturrialba* specimens captured by month (2001–2006).

A total of 81 specimens of *Xystromutillaturrialba* (79 males and 2 females) were captured over six continuous sampling years (2001–2006) with ten Malaise traps on Barro Colorado Island (BCI), Panama. The years with the greatest abundance of specimens were 2001 and 2003; samples were not captured during 2005 and 2006 (Fig. 9A). Specimens of *X.turrialba* were captured only in the months from February to July, with greatest abundance from March to May with 71 (87.6%) specimens captured, peaking during April with 44 specimens (54.3%) (Fig. 9B). The greater abundance of *X.turrialba* during April is similar to results found for *Dasymutilla* Ashmead, 1899 and *Ephuta* Say, 1836 (Mutillidae) species on BCI (Cambra et al. 2018; Añino et al. 2020). The only two females of *X.turrialba* were captured in March and April, and their relatively small number compared to that of males on BCI is due to the sampling methodology, since females are apterous. The under-representation of females is not seen in hand-net and trap-nest samples from Panama, in which 14 females and 16 males of *X.turrialba* were captured during all months of the year except November (Cambra and Quintero 1992, 2004).

## Discussion

Both sexes of *Xystromutillabucki* are morphologically similar to some species of the genus *Sphaeropthalma* Blake, 1871, especially those recorded from Central and South America (Williams and Pitts 2007). Brothers (2006) presented a key for the Neotropical genera of Mutillidae, placing both *Xystromutilla* and *Sphaeropthalma* in the same couplet and separating them based on the presence or absence of plumose setae on the head, as well as the shape of the first metasomal tergum. It is important to note that *Sphaeropthalma* is a sort of “dumping ground” genus for nocturnal species of velvet ants in South America and many of its species will likely be placed in different genera in the future (KAW, PRB and RAC, pers. obs.). Sexual associations are of great importance to understand the phylogenetic relationships of *Xystromutilla*, *Sphaeropthalma* and any other genera to be dismembered from the latter, as well as to have a better knowledge of the morphological characteristics that delimit Sphaeropthalmini genera in the New World.

Little information exists on overwintering/diapause in Mutillidae. Mickel (1928) summarized earlier information from several sources in compiling a typical mutillid life history and stated that “in colder latitudes the winter is passed in the prepupal stage”. Bohart and McSwain (1939) mentioned prepupal overwintering for *Dasymutillasackenii* (Cresson, 1865) from California. Brothers (1972, 1978) observed diapause in the fifth larval instar (prepupa) of some *Pseudomethocafrigida* (Smith, 1855) and *Myrmosulaparvula* (Fox, 1893) kept in the laboratory. Brothers (1972) also presented a summary of unpublished observations by Cottrell, indicating that some individuals of *Dasymutillabioculata* (Cresson, 1865) may diapause as prepupae for more than one season. Our finding of prepupal diapause in *Xystromutillabucki* represents the first record for a Neotropical mutillid species and reinforces the apparently general occurrence of such diapause where environmental conditions are appropriate.

**Figures 10, 11.**
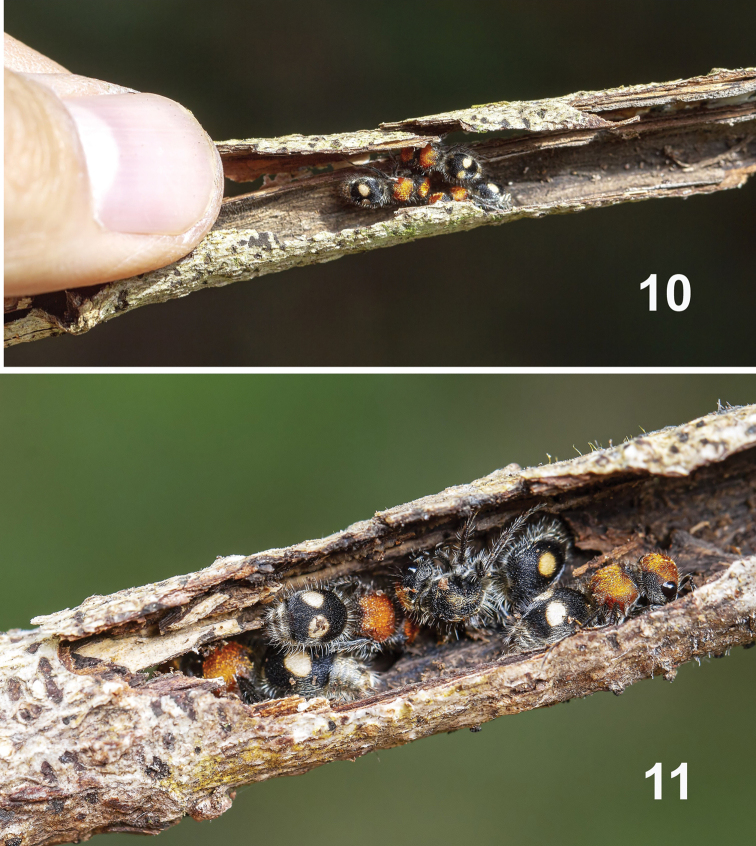
Pseudomethocanr.chontalensis inside of hollow *Quercusinsignis* twig (manually opened).

Apart from prepupal diapause, there are a few records of hibernation/diapause by adults. Potts and Smith (1944) recorded hibernation in two adult females of *Dasymutillaaureolapacifica* (Cresson, 1875) from California; Evans and Miller (1970) indicated some degree of overwintering by three adult females (marked the previous summer of 1968) of *D.nigripes* (Fabricius, 1787) collected in the summer of 1969 in Michigan; and Hennessey (2002) recorded overwintering by adult females of *D.nigripes* (Fabricius), *D.vesta* (Cresson, 1865) and *Timullavagans* (Fabricius, 1798) in a deciduous forest in Maryland. The following observations on Pseudomethocanr.chontalensis (Cameron, 1895), det. R. Cambra, by Kenji Nishida (unpublished data) are also relevant here. On January 25, 2015, fifteen adult females of P.nr.chontalensis (with two dark morphs) were found inside a hollow and dry twig of *Quercusinsignis* (Fagaceae) (Figs 10, 11). The twig was found on the ground in a relatively open area of the forest in EBM, Monteverde at 1530 m, Pacific slope, Costa Rica; the weather this time of the year is windy and cold, and the temperature was no higher than 16 °C during the morning or afternoon and 12 °C during the night. The twig on the ground used as ‘shelter’ was an old, empty, cut-off branch made by a female Cerambycidae (Coleoptera) larva (det. K. Nishida), which had probably eaten much of the interior of the twig. No traces or remains of larvae, prepupae, or pupae of any other insect were found, suggesting that the mutillids did not emerge inside the twig. When the twig was manually opened, the 15 mutillids were observed well grouped, with some on top of the others. Two fell to the ground and escaped into the litter; the other 13 (three groups, of six, four, and three specimens) remained motionless within the twig. On February 5, 2015, all the females began to move and left the twig. The temperature was between 24 and 28 °C. The 13 females remained grouped for 12 days. This immobile aggregation indicates a probable diapause in adult females of P.nr.chontalensis. Janvier (1933: 283) recorded a single male in the middle of a group of 46 female mutillids captured in Chile (identified as *Dimorphomutillaformosa* Mickel, 1938 by Quintero and Cambra 2001). Janvier did not mention how long they were grouped. We do not know of another record related to possible diapause in adults of Neotropical Mutillidae.

Mutillids are solitary wasps; in tropical forests they are generally widely dispersed. We do not know how P.nr.chontalensis females would form such an aggregation. However, females and males of Mutillidae produce sounds that may be species specific (Tschuch 1993; Torrico-Bazoberry and Muñoz 2019). The function of stridulation in mutillids is unclear, although these sounds apparently act as warning/defensive signals (Schmidt and Blum 1977; Masters 1979; Polidori et al. 2012) and/or have roles in intra-specific communication (Bayliss and Brothers 1996; Torrico-Bazoberry and Muñoz 2019), but there is no evidence suggesting that such signals could be used in forming aggregations. Although we cannot definitively know the function of aggregation in P.nr.chontalensis, we suggest that it is a defensive strategy against potential predators while waiting for favorable environmental conditions for dispersal, mating and host searching activities.

## Supplementary Material

XML Treatment for Xystromutilla bucki

XML Treatment for Xystromutilla turrialba

## References

[B1] André E (1905) Nouvelles espèces de Mutillides d’Amérique. (Hym.). Zeitschrift für Systematische Hymenopterologie und Dipterologie 5: 361–376.

[B2] André E (1906) Nouvelles espèces de Mutillides d’Amérique (Hym.). Zeitschrift für Systematische Hymenopterologie und Dipterologie 6: 33–48. [65–80, 161–169.]

[B3] Añino YJ Cambra RA Windsor DM Williams KA Bartholomay PR Quintero D Sánchez V (2020) Seasonal and annual variation in the abundance of *Ephuta* Say (Hymenoptera: Mutillidae) in Panama. Revista de Biología Tropical 68(2): 573–579.

[B4] Ashmead WH (1899) Superfamilies in the Hymenoptera and generic synopses of the families Thynnidae, Myrmosidae, and Mutillidae. Journal of the New York Entomological Society 7: 45–60. https://www.biodiversitylibrary.org/page/9198707

[B5] Bartholomay PR Williams KA Cambra RA Oliveira ML (2019) Does the genus *Dasymutilla* Ashmead occur in South America? The new genus *Quwitilla*, new combinations, and new distribution records from Neotropical velvet ants (Hymenoptera: Mutillidae). Zootaxa 4623(2): 261–282. 10.11646/zootaxa.4623.2.3 31716260

[B6] Bayliss PS Brothers DJ (1996) Biology of *Tricholabiodes* Radoszkowski in southern Africa, with a new synonymy and review of recent biological literature (Hymenoptera: Mutillidae). Journal of Hymenoptera Research 5: 249–258.

[B7] Blake CA (1871) Synopsis of the Mutillidae of North America. Transactions of the American Entomological Society 3: 217–265. 10.2307/25076249

[B8] Bohart GE McSwain JW (1939) The life history of the sand wasp, *Bembixoccidentalisbeutenmuelleri* Fox and its parasites. Bulletin of the Southern Academy of Sciences 38: 84–97.

[B9] Brothers DJ (1972) Biology and immature stages of *Pseudomethocaf.**frigida*, with notes on other species (Hymenoptera: Mutillidae). The University of Kansas Science Bulletin 50(1): 1–38.

[B10] Brothers DJ (1978) Biology and immature stages of *Myrmosulaparvula* (Hymenoptera: Mutillidae). Journal of the Kansas Entomological Society 51(4): 698–710.

[B11] Brothers DJ (2006) Mutillidae. In: Hanson P Gauld I (Eds) Hymenoptera de la Región Tropical.Memoirs of the American entomological Institute 77: 586–594.

[B12] Brothers DJ Lelej AS (2017) Phylogeny and higher classification of Mutillidae (Hymenoptera) based on morphological reanalyses. Journal of Hymenoptera Research 60: 1–97. 10.3897/jhr.60.20091

[B13] Cambra RA Quintero D (1992) Velvet ants of Panama: distribution and systematics (Hymenoptera: Mutillidae). In: Quintero D Aiello A (Eds) Insects of Panama and Mesoamerica: Selected Studies.Oxford University Press, Oxford, 459–478.

[B14] Cambra RA Quintero D (2004) New species of *Xystromutilla* André (Hymenoptera: Mutillidae) and the first illustrated key for the males of the genus. Transactions of the American Entomological Society 130(4): 463–478.

[B15] Cambra RA Tunes-Buschini ML Quintero D Brozoski F Rudiak-Lustosa P (2017) *Ephutaicema* Casal, 1969 and its host *Auplopussubaurarius* Dreisbach, 1963 (Hymenoptera: Mutillidae, Pompilidae) from Brazil. Zootaxa 4272(2): 285–290. 10.11646/zootaxa.4272.2.9 28610297

[B16] Cambra RA Williams KA Quintero D Windsor D Pickering J Saavedra D (2018) *Dasymutilla* Ashmead (Hymenoptera, Mutillidae) in Panama: new species, sex associations and seasonal flight activity. Insecta Mundi 0608: 1–17.

[B17] Cameron P (1894–1896) Biologia Centrali-Americana. Hymenoptera 2: 259–395.

[B18] Casal OH (1969) Sobre *Xystromutilla* André, 1905 (Hymenoptera, Mutillidae). Physis 29: 47–50.

[B19] Cresson ET (1865) Descriptions of some new species of *Mutilla* from California. Proceedings of the Entomological Society of Philadelphia 4: 385–390. https://www.biodiversitylibrary.org/page/3328793

[B20] Cresson ET (1875) Descriptions of new species of *Mutilla*. Transactions of the American Entomological Society 5: 119–120. 10.2307/25076298

[B21] Dreisbach RR (1963) New species of spider wasps, genus *Auplopus*, from the Americas South of the United States (Hymenoptera: Psammocharidae). Proceedings of the United States National Museum 114(3468): 137–211. [13 pls] 10.5479/si.00963801.114-3468.137

[B22] Evans DA Miller BR (1970) A note on adult overwintering of *Dasymutillanigripes* in Michigan (Hymenoptera: Mutillidae). The Michigan Entomologist 2(3–4): 74–74.

[B23] Fabricius JC (1787) Mantissa Insectorum, sistens eorum nuper detecta, adjectis characteribus genericus, differentiis specificus, emendationibus, obserevationibus. Hafniae, Proft. 1: 311–313. 10.5962/bhl.title.11657

[B24] Fabricius JC (1798) Supplementum Entomologiae Systematicae. t. 5, 42–42. https://www.biodiversitylibrary.org/page/42138538

[B25] Fabricius JC (1804) Systema Piezatorum secundum ordinis, genera, species, adjectis synonymis, locis, observationibus, descriptionibus. Brunswick: C. Reichard, [xiv + 15–439 + 30] 469 pp. 10.5962/bhl.title.10490

[B26] Fox WJ (1893) New North American Aculeate Hymenoptera. Journal of the New York Entomological Society 1: 53–53. https://www.biodiversitylibrary.org/page/33454707

[B27] Hennessey RD (2002) Population-level characteristics of *Dasymutillanigripes*, *D.vesta*, and *Timullavagans* (Hymenoptera: Mutillidae). Florida Entomologist 85(1): 245–253. 10.1653/0015-4040(2002)085[0245:PLCODN]2.0.CO;2

[B28] Janvier H (1933) Étude biologique de quelques hyménoptères du Chili. Annales des sciences naturelles. Zoologie et biologie animale (Ser. 10) 16: 209–356.

[B29] Masters WM (1979) Insect disturbance stridulation: its defensive role. Behavioral Ecology and Sociobiology 5(2): 187–200. 10.1007/BF00293305

[B30] Mickel CE (1928) Biological and taxonomic investigations on the mutillid wasps. Bulletin of the United States National Museum 143: 1–360. [+ 5 pls] 10.5479/si.03629236.143.1

[B31] Mickel CE (1938) The Neotropical mutillid wasps of the genus *Dimorphomutilla* Ashmead (Hymenoptera). Revista de Entomologia, Rio de Janeiro 9: 349–364.

[B32] Morato EF (1994) *Xystromutillaasperiventris* André, 1905 (Mutillidae) reared from sphecid wasps in trap-nests, Manaus Amazonas, Brazil. Sphecos 28: 13–14.

[B33] Pagliano G Brothers DJ Cambra RA Lelej AS Lo Cascio P Matteini-Palmerini M Scaramozzino PL Williams KA Romano M (2020 [“2018”]) Checklist of names in Mutillidae (Hymenoptera), with illustrations of selected species. Bollettino del Museo Regionale di Scienze Naturali di Torino 36(1–2): 5–427.

[B34] Polidori C Ruffato G Borruso L Settanni C Pavan G (2012) [“2013”] Stridulatory organ and distress call in males and females of a small velvet ant (Hymenoptera: Mutillidae). Bioacoustics 22(2): 121–135. 10.1080/09524622.2012.736241

[B35] Potts RW Smith RF (1944) Hibernation of *Dasymutillaaureolapacifica*. The Pan-Pacific Entomologist 20(2): 60–60.

[B36] Quintero D Cambra RA (2001) On the identity of *Scaptopoda* F. Lynch Arribálzaga, new taxonomic changes and new distribution records for Neotropical Mutillidae (Hymenoptera), with notes on their biology. Transactions of the American Entomological Society 127(3): 291–304.

[B37] Richards OW (1934) The American species of the genus *Trypoxylon* (hymenopt., sphecoidea). Transactions of the Royal Entomological Society of London 82(2): 173–362. 10.1111/j.1365-2311.1934.tb00033.x

[B38] Rodríguez AR Matías FA (1996) Diversidad de himenópteros usuarios de trampas-nidos, sus parasitoides y sus preferencias de anidación en Península Gigante. Thesis, Universidad de Panamá.

[B39] Saussure H de (1867) Hymenoptera. In: Reise der östterreischen Fregatte Novara um die Erde. Zoologischer Theil 2: 1–138.

[B40] Say T (1836) Descriptions of new species of North American Hymenoptera, and observations on some already described. Boston Journal of Natural History 1(3): 295–298. https://www.biodiversitylibrary.org/page/32414019

[B41] Schmidt JO Blum MS (1977) Adaptations and responses of *Dasymutillaoccidentalis* (Hymenoptera: Mutillidae) to predators. Entomologia Experimentalis et Applicata 21(2): 99–111. 10.1111/j.1570-7458.1977.tb02663.x

[B42] Smith F (1855) Catalogue of hymenopterous insects in the collection of the British Museum, part III. Mutillidae and Pompilidae. London, 63 pp. https://www.biodiversitylibrary.org/page/9319972

[B43] Smith F (1856) Catalogue of hymenopterous insects in the collection of the British Museum, part IV, Sphecidae, Larridae and Crabronidae. London, 207–497. 10.5962/bhl.title.20858

[B44] Suárez FJ (1960) Datos sobre mutílidos neotropicales I. Nuevas especies de Sphaerophthalminae (!) (Hymenoptera). EOS 36(4): 451–480.

[B45] Torrico-Bazoberry D Muñoz MI (2019) High-frequency components in the distress stridulation of Chilean endemic velvet ants (Hymenoptera: Mutillidae). Revista Chilena de Entomología 45(1): 5–13.

[B46] Tschuch G (1993) Sound production in mutillid wasps (Mutillidae, Hymenoptera). Bioacoustics 5(1–2): 123–129. 10.1080/09524622.1993.9753234

[B47] Williams KA Pitts JP (2007) New species of the predominately temperate velvet ant genera *Lomachaeta* Mickel and *Sphaeropthalma* Blake from Central and northern South America (Hymenoptera: Mutillidae). Transactions of the American Entomological Society 133(3+4): 297–326. 10.3157/0002-8320-133.3.297

